# Control of multilayer biological networks and applied to target identification of complex diseases

**DOI:** 10.1186/s12859-019-2841-2

**Published:** 2019-05-28

**Authors:** Wei Zheng, Dingjie Wang, Xiufen Zou

**Affiliations:** School of Mathematics and Statistics, Wuhan University, Wuhan, 430072 China

**Keywords:** Multilayer networks, Network control, Nonlinear dynamical systems, IISG algorithm, Driver nodes

## Abstract

**Background:**

Networks have been widely used to model the structures of various biological systems. The ultimate aim of research on biological networks is to steer biological system structures to desired states by manipulating signals. Despite great advances in the linear control of single-layer networks, it has been observed that many complex biological systems have a multilayer networked structure and extremely complicated nonlinear processes.

**Result:**

In this study, we propose a general framework for controlling nonlinear dynamical systems with multilayer networked structures by formulating the problem as a minimum union optimization problem. In particular, we offer a novel approach for identifying the minimal driver nodes that can steer a multilayered nonlinear dynamical system toward any desired dynamical attractor. Three disease-related biology multilayer networks are used to demonstrate the effectiveness of our approaches. Moreover, in the set of minimum driver nodes identified by the algorithm we proposed, we confirmed that some nodes can act as drug targets in the biological experiments. Other nodes have not been reported as drug targets; however, they are also involved in important biological processes from existing literature.

**Conclusions:**

The proposed method could be a promising tool for determining higher drug target enrichment or more meaningful steering nodes for studying complex diseases.

**Electronic supplementary material:**

The online version of this article (10.1186/s12859-019-2841-2) contains supplementary material, which is available to authorized users.

## Background

Biological processes, which are indispensable for living organisms, are often carried out by complex interactions among various biological elements. Studying biological elements and their interactions is vital for understanding the roles of intracellular biomolecules and the mechanisms of biological processes. The structure of biological systems can be modeled by biological networks in which nodes are biological elements and edges connect biological elements that interact with one another. Therefore, networks have been widely used to model the structures of various biological systems. Moreover, the ultimate aim of research on biological networks is to steer the states of biological systems to desired states by manipulating signals. Recently, controllability, which is a concept in control theory, has been applied to investigate the dynamics of complex networks.

In the past few decades, the controllability of single-layer networks with linear [1–5] and nonlinear [6–9] dynamics have been widely studied in a variety of biomedical systems. In particular, an innovative study of the newly developed linear control approaches is a structural controllability framework [2], that involves using a graph-based tool to identify the minimal driver nodes set by formulating this problem as a maximum matching problem. Then, many other methods related to linear control of single-layer networks were proposed [1, 5] and some important topics in network controllability, including observability [10], control energy [3, 11, 12], target control [13], etc., were discussed. With a deeper realization of network controllability, many researchers have observed that multiple real-world complex systems are extremely complicated nonlinear processes. Therefore, several new approaches have been proposed for controlling nonlinear single-layer networks [6–9]. In particular, a breakthrough in recently developed methods led to mapping the controllability of a single network to the feedback vertex set (FVS) problem in which the objective is to drive the nonlinear single networked system from an arbitrary initial state to any desired dynamical attractor (e.g., a steady state) by overriding the state of a certain node [8, 14]. In our recent study [15, 16], we established a new theory of domain controllability to describe the controllability of nonlinear dynamical networks and demonstrated how to drive a complex networked system in transition from the attraction domain of a stable steady state to the attraction domain of another stable steady state.

However, with the development of network controllability theory, it has been shown that biological networks are always governed by multiple types of interactions or interact with other networks. Single networks might be insufficient to discover the underlying biological mechanism. For example, cellular activities in biochemical networks, gene regulatory networks, and a biochemical reactions network are all highly interdependent and are excellent choices for being analyzed as multilayer networks [17–19]. In recent years, a multilayer network method has been proposed to describe such complex, multidimensional biological systems. A multilayer network can link human diseases with genetic, biochemical, and environmental factors [20–23].

More recently, some advances [24–27] have focused on the linear control of multilayer networks. Menichetti et al. [25] proposed a combinatorial matching model to identify the minimal set of driver nodes in multilayer networks. Pósfai et al. [26] developed a theory based on disjoint path covers to determine the minimum number of inputs necessary for full control of multiplex, multiscale networks. Recent efforts have dedicated to understanding the interplay between the degree correlation of interconnections and the controllability of multilayer networks [27, 28].

However, from the perspective of a dynamical process, many biological systems are extremely complicated nonlinear dynamical processes such as cell signaling, information transmission [8, 29, 30]. Exploring the controllability properties of these real-world complex systems has fundamental importance and multiple applications in biological contexts [31, 32]. Until now, there has been a lack of a suitable approach for controlling nonlinear dynamical systems with multilayer networked structures. Determining how to control a nonlinear multilayer networked system still is a crucial and challenging topic.

In this study, we focus on nonlinear multilayer networked systems, which consist of a fixed set of nodes connected by different types of links, and each layer has a complicated nonlinear dynamical process. To explore a general framework for controlling nonlinear multiplayer networked systems, we propose a novel strategy for formulating the problem as a minimum union optimization problem. In particular, we offer a novel approach for identifying the minimum set of driver nodes that steer a multilayered nonlinear dynamical system toward any desired dynamical attractor. Three disease-related biological multilayer networks are used to illustrate the effectiveness of our proposed approach. We discovered that in the minimal set of driver nodes, which were identified using the proposed algorithm, some nodes could act as drug targets in biological experiments. Other nodes were also demonstrated to be involved in important biological processes from the existing literature.

## Results

**Fig. 1:**
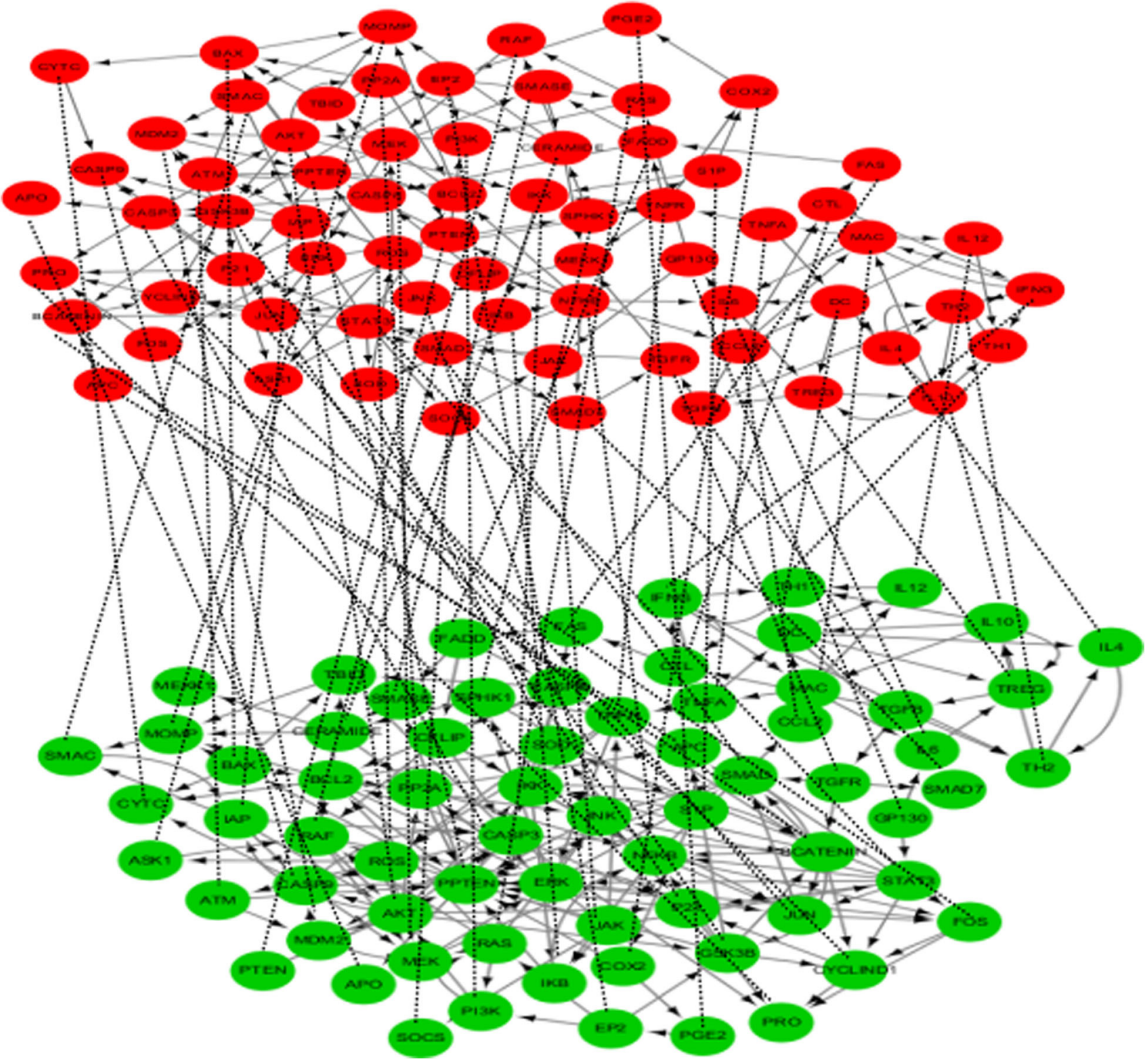
Colitis-Associated Colon Cancer (CACC) network**.** The first layer contains 70 nodes and 154 regulatory interactions and the second layer has the same 70 nodes and different 200 different links

**Fig. 2:**
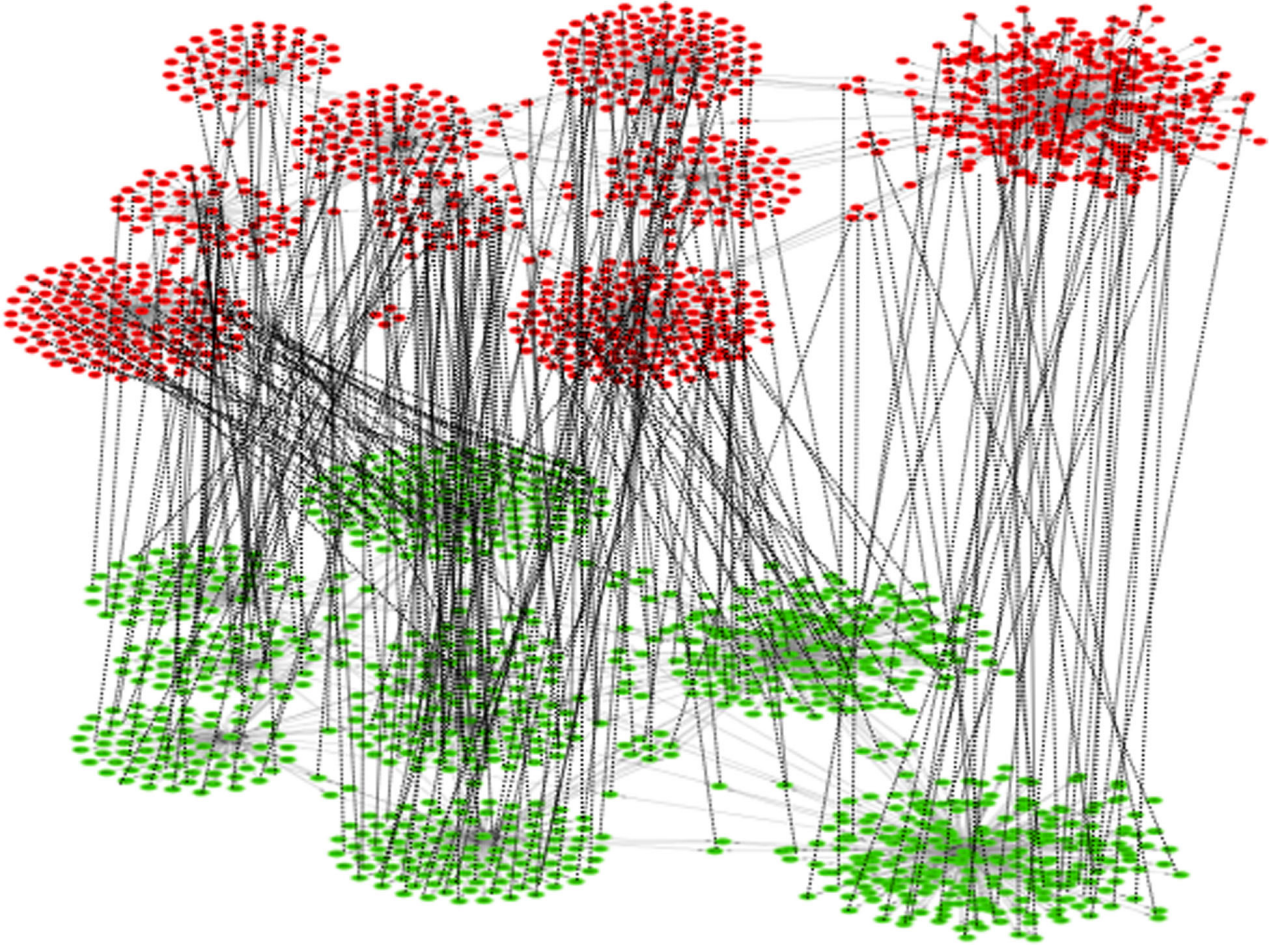
Human-HIV1 Multiplex Gpi (HHMG) network. The first layer represents physical associations with 1157 edges and 1004 nodes. The second layer represents direct interactions with 1135 edges and 1004 nodes. The interaction relationships are derived from the BioGRID database

**Fig. 3:**
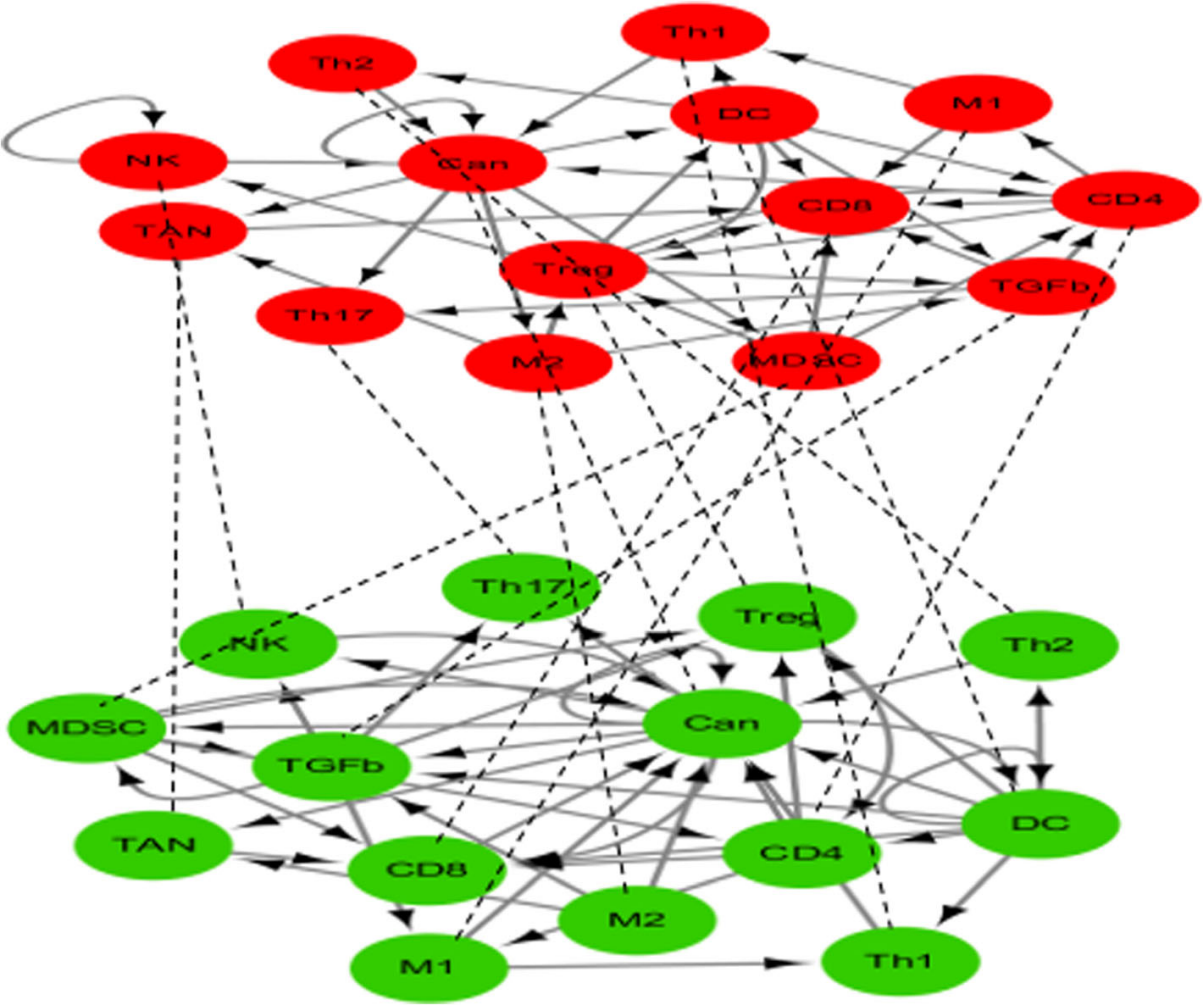
Cancer-Immune Cell–Cell (CICC) interaction network. The first layer has 14 nodes and 38 links, and the second layer contains the same 14 nodes and 42 regulatory interactions

We investigated the minimal feedback vertex set (MFVS) of three regulatory multilayer networks, including the Colitis-Associated Colon Cancer (CACC) network, Human-HIV1 Multiplex Gpi (HHMG) network and Cancer–Immune Cell–Cell (CICC) interaction network (Figs. 1, 2 and 3, respectively). We demonstrated that our proposed method is capable of determining the MFVS of networks such that the steering nodes in the MFVS can steer a multilayer nonlinear dynamical system. The data sources used in constructing multilayer networks are shown in Table 1. The identified driver nodes for these three networks are listed in Table 2, and their detailed functions are shown in Additional file 1: Table S1.

**Table 1:** Data sources for three multilayer networks: CACC, HHMG and CICC networks

CACC network		HHMG network		CICC network	
First layer	Second layer	First layer	Second layer	First layer	Second layer
CAC network [34]	DirectedPPI database +KEGG database+ CAC network [34]	CoMuNe lab (https://comunelab.fbk.eu/data.php)	CoMuNe lab (https://comunelab.fbk.eu/data.php)	CIC network [47]	CIS network [48]

**Table 2:** Identified driver nodes for three multilayer networks: CACC, HHMG and CICC networks

CACC network (17)		HHMG network (9)	CICC network (5)
AKT	RAF	ENV	TGFß
P21	SMAD	GAG-POL	Cancer cells
BCATENIN	P53	GAG	NK cells
IFNG	IAP	NEF	CD 4 T cells
JAK	CASP9	VPU	DC
JUN	IL4	REV	
NFKB	SPHK1	VIF	
IKB	TREG	VPR	
PI3K		TAT	

### Colitis-Associated Colon Cancer (CACC) network

F. Balkwill et al. [33] discovered that inflammation and cancer are closely related. Lu et al. [34] investigated inflammation-associated cancer and constructed a CAC network with 70 nodes and 154 regulatory interactions by exploring the GeneGo database.

To deeply understand the mechanisms of inflammation and cancer, we integrate colon cancer data from different sources, including the DirectedPPI database (www.flyrnai.org/DirectedPPI), KEGG database (www.kegg.jp/) and the work of Lu et al. [34] to build a duplex network (Fig. 1). The data sources are shown in the first column of Table 1.

Applying our method to the CACC network, we identify the two-layer MFVS, which can drive this multilayer networked system from an arbitrary initial state to any desired dynamical attractor by providing proper external signals. There are 17 steering nodes, including AKT, CASP9, P21, BCATENIN, IFNG, IL4, JAK, JUN, NFKB, IKB, PI3K, RAF, SMAD, SPHK1, P53, TREG and IAP in the identified MFVS. Among these nodes, there a 13 steering nodes, including AKT, P21, BCATENIN, IFNG, JAK, JUN, NFKB, IKB, PI3K, RAF, SMAD, P53 and IAP, that are drug targets and each node can interact with 5.00 drugs on average according to the DrugBank database (https://www.drugbank.ca) [35]. The different names for the drugs are listed in Additional file 1: Table S2.

Though SPHK1, TREG, CASP9 and IL4 cannot be considered as drug targets, they are also involved in important biological processes. SPHK1 plays an important role in tumorigenesis, hormonal therapy, chemotherapy resistance, and it is considered to be a new target for cancer therapeutics [36]. TREG is a type of regulatory T cell that is one of the major components for immunosuppression and promotes suppressive cytokines as well as inhibiting effector T cells (CD8 T cells and NK cells) directly in the cancer-immune system [37]. Based on the STITCH database (http://stitch.embl.de/) [38], CASP9 can interact with 4 chemicals, namely, cisplatin, 15d-PGJ2, cordycepin and hydrogen peroxide. Of these chemicals, cisplatin is a platinum-based chemotherapy drug used to treat various types of cancers, including sarcomas, some carcinomas (e.g., small cell lung cancer and ovarian cancer), lymphomas and germ cell tumors. IL4 can interact with 4 chemicals, namely, montelukast, ALLERGENS, retinoic acid, and tacrolimus. Tacrolimus is an immunosuppressive drug and retinoic acid is also a medication used for the treatment of some certain cancers. Therefore, we believe SPHK1, TREG, CASP9 and IL4 can be regarded as potential drug targets that are critical for the treatment of colon cancer.

If we only consider a single-layer network, i.e., the CAC network with the SG algorithm, the driver nodes we obtained represented 10 nodes, including AKT, P21, IFNG, IL4, IKB, SMAD, P53, TREG, MEK and STAT3. 7 Proteins AKT, P21, IFNG, IKB, SMAD, P53 and MEK are demonstrated as drug targets from DrugBank database. Moreover, we compared the proportion of drug targets for the different driving nodes (Table 3). Compared to single-layer network (0.7), a multilayer network (0.72\0.77) obtained driver nodes with higher target proportions. These results show that by integrating different interaction relations, multilayer networks can describe biological processes more accurately than single-layer networks, and more significant results can be obtained from them. In particular, the steering nodes with the IISG algorithm are more enriched with known drug targets (0.77), which support the applicability of the method.

**Table 3:** Comparisons of the proportion of drug targets using three different methods, including SG, ISG and the proposed IISG for the CAC and CACC networks

	Proportion of Drug Targets
SG algorithm with CAC network	0.7
ISG algorithm with CACC network	0.72
IISG algorithm with CACC network	0.77

**Fig. 4:**
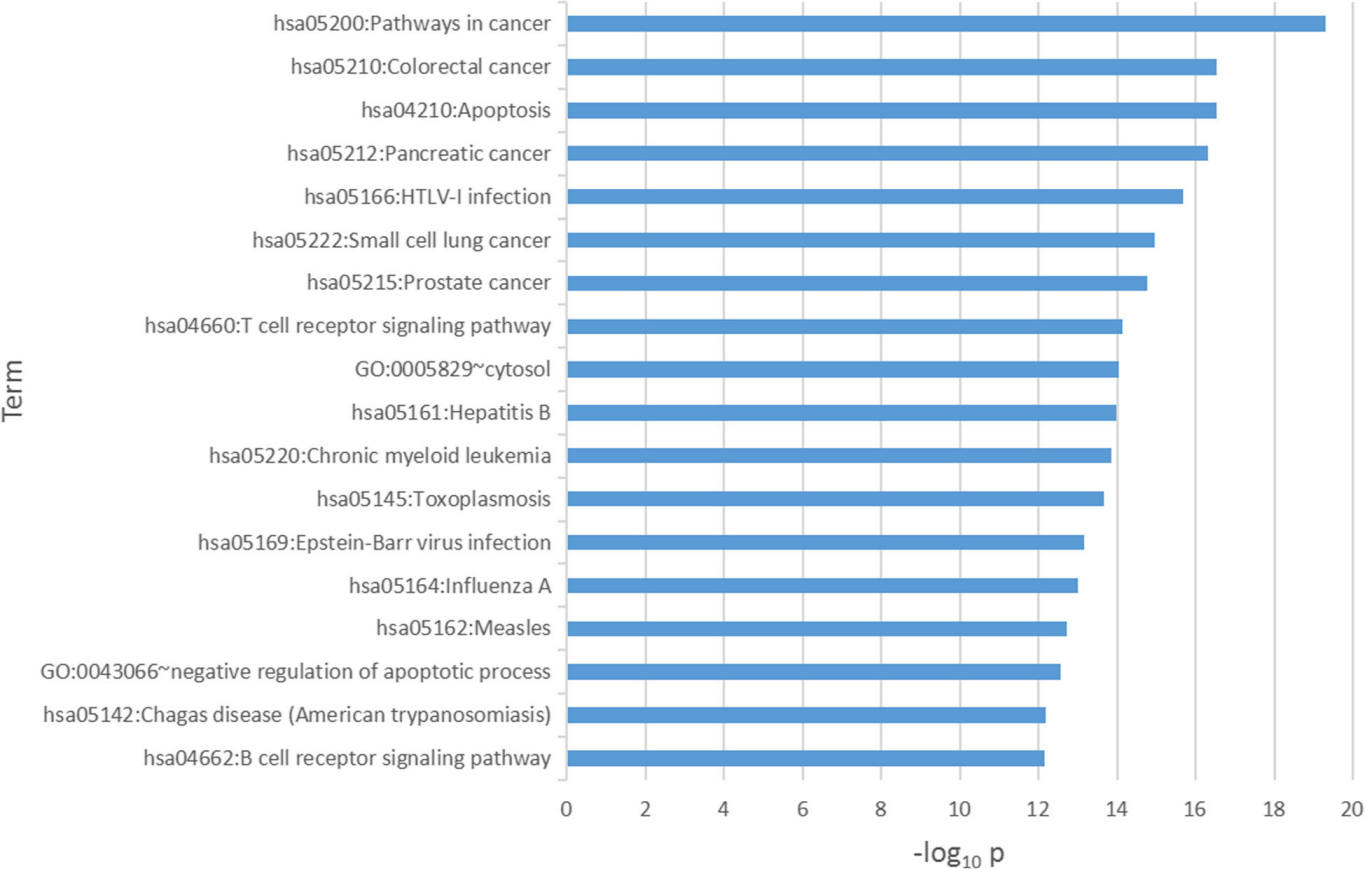
The 18 significant pathways of genes in CACC network and p values are less than 7.00E-13. The p values of pathways in cancer and Colorectal cancer pathway are minimal

Based on DAVID Database (https://david.ncifcrf.gov/), the 18 significant pathways of genes in CACC network that p values are less than 7.00E-13 are shown in Fig. 4 and Additional file 1: Figures S1-S5. From Fig. 4 and Additional file 1: Figures S1-S5, we can observe that identified driver nodes are enriched in T cell and B cell receptor signaling pathways and apoptotic process, which are three of eight hallmarks of cancer. Moreover, we obtained the top 10 genes in pathways through sorting the p values from small to large by using MSigDB Database (http://software.broadinstitute.org/gsea/msigdb). Additional file 1: Figure S6 illustrates that whether a gene belongs to a particular pathway clearly. Specifically, in “Pathways in cancer” and “Colorectal cancer pathway” with the minimal p values, we discovered the same module of “PI3K-AKT signaling pathway”, “MAPK signaling pathway” and “NF-κB signaling pathway” in these two pathways (Additional file 1: Figures S1-S5), which could provide a novel research direction on colon cancer treatments.

### Human-HIV1 Multiplex Gpi (HHMG) network

Human Immunodeficiency Virus (HIV) is one of the most notorious viruses that humans have ever faced. Despite many HIV studies conducted over 30 years, the mechanism of HIV remains poorly understood. Researchers found that the infection processes of HIV-1 are related to different types of genetic interactions, which can be represented by a multiplex network (https://comunelab.fbk.eu/data.php). We integrated this HHMG network into a two-layer duplex network (Fig. 2) in which the first layer represents a physical association with 1157 edges and 1004 nodes. The second layer represents direct interaction with 1135 edges and 1004 nodes. The interaction relationships are derived from the BioGRID database and literature.

Applying the IISG algorithm to the HHMG network, we identified the MFVS with 9 steering nodes, including VPR, TAT, ENV, GAG-POL, GAG, NEF, VPU, REV and VIF, which are shown in the second column of Table 2. More importantly, four proteins, including ENV, GAG-POL, GAG and NEF, were experimentally validated to be drug targets in the DrugBank Database (Additional file 1: Table S1). The different names for the drugs are listed in Additional file 1: Table S3. Moreover, another five nodes, which are VPR, TAT, VPU, REV and VIF, were also involved in important biological processes. HIV-1 encodes VPR, which is a 96-amino-acid protein that can prevent proliferation of infected cells by acting primarily as a cytostatic drug [39]. It has been revealed that the mechanism of TAT activation involves RNA Polymerase II elongation of the integrated HIV-1 [40]. VPU, which is an accessory protein that is encoding by HIV-1, is involved in several immunomodulatory functions, including counteraction of the host restriction factor tetherin and down modulation of CD4 T cells [41]. In addition to controlled processing of RNA, HIV-1 replication is also dependent on the activities provided by TAT and REV encoding by HIV-1 [40]. VIF, HIV-1 accessory protein is necessary for the production of infectious virions by CD4 lymphocytes [42] Moreover, according to the STITCH Database, VPR, TAT, REV and VIF can interact with 1, 5, 2, 4 kinds of chemicals, respectively. Therefore, VPR, TAT, VPU, REV and VIF can be considered potential drug targets for treating the HIV-1.

To further demonstrate the advantages of using multilayer network, we compared the results using only a single-layer network and multilayer network. Table 4 lists the driver nodes using the first layer network (DNL1), the second layer network (DNL2) and a network with two layers (DNL3). From Table 4, we can observe that the main difference is the obtained protein ENV, which is an important drug target because it can combine with 8 kinds of drugs according to DrugBank (Additional file 1: Table S3). We also analyzed the gene enrichment of genes in MFVS of the HHMG network. From the results in Additional file 1: Table S4, we can see that these genes are involved in host-virus interactions, which are agreement with the experimental evidences.

**Table 4:** Comparisons of identified driver nodes using single-layer and multilayer networks for the HHMG network

DNL1	VPR	TAT	ENV		GAG-POL	GAG	NEF	VPU		VIF
DNL 2	VPR	TAT		CD 4 T cells	GAG-POL	GAG	NEF	VPU	REV	VIF
DNL 3	VPR	TAT	ENV		GAG-POL	GAG	NEF	VPU	REV	VIF

### Cancer–Immune Cell–Cell (CICC) interaction network

Immune cells have been suggested to play paramount roles in controlling malignancy [43, 44]. Recently, large efforts have been devoted to cancer immunotherapy by investigating the cancer–immunity interaction network [45, 46]. Li [47] mapped the complicated interactions among cancer cells and immune cells onto a cancer–immunity cell–cell interaction network (CIC network) including 10 nodes (9 representative cell types and an important cytokine) and 28 interaction links to explore the biological principles between cancer and immunity. Similarly, Wang et al. [48] constructed a comprehensive cancer–immune system (CIS network) including 26 nodes (13 representative cell types and 13cytokine) and 107 interaction links by collecting data from the existing literature.

To deeply understand the mechanisms between the cancer and the immune system, we integrate the CIC network and CIS network to build a duplex network (Fig. 3). The data sources are shown in the third column in Table 1. Applying the IISG algorithm to the CICC network, we identified the MFVS for two layers with five nodes, which was reduced by one (“TREG”) compared to the ISG algorithm (see the last row in Table 5). To further demonstrate the advantages of using a multilayer network, we compare the results using only a single-layer network and multilayer network. Table 5 lists the driver nodes using the first layer network (DNL1), the second layer network (DNL2) and a network with two layers (DNL3). From Additional file 1: Table S5, we can see that the identified TGFß is considered to be a critical suppressor of T cell activities which kill the tumor cells directly [37]. TGFß leads to cancer proliferation [49]. Cancer cells and natural killer (NK) cells have a self-growth system. Cancer cells are the major target we would like to investigate and the NK cells, by boosting the immune system and restricting the growth of tumors, reconsidered to be one of the major inhibitors of tumor cells. Increasing the proliferation of NK cells is an anticancer strategy [47]. By promoting the activity of CD8, CD4 and TREG cells, dendritic cells (DC) provide major mechanisms for T cell activation. CD4 T cells are one of the inhibiting cell types for cancer. CD4 T cells are an equally critical component for the antitumor immune response. Successful immunity to cancer therefore requires activation of tumor-specific CD4 T cells [50]. In Additional file 1: Table S5, we display the detailed biological information of cytokines and cell types related to cancer immune system. To sum up, these results demonstrated that in cancer-immune related cell types and cytokines, five nodes including four cell types (Cancer cells, NK cells, DC and CD4 T cells) and one cytokine (TGFß), are critical for discovering the biological principles and insights that clarify the interplay between cancer and immunity, and these nodes are identified targets for cancer immunotherapy.

**Table 5:** Comparisons of identified driver nodes using single-layer and multilayer networks for the CICC network

DNL1		Cancer cells	NK cells			TREG
DNL2	TGFß	Cancer cells		CD 4 T cells	DC	
DNL3	TGFß	Cancer cells	NK cells	CD 4 T cells	DC	

## Discussion

In this study, we investigated the nonlinear control of multilayer networks by proposing a novel approach, the IISG algorithm, for identifying the minimal set of driver nodes that steer a multilayered nonlinear dynamical system toward any desired dynamical attractor.

We applied the algorithm to three real biological networks, including the CACC, HHMG and CICC networks. The IISG algorithm is capable of obtaining the minimal FVS of a duplex network compared to the ISG algorithm. Through comparisons of the results using a single-layer network and a multilayer network, its effectiveness are demonstrated. In future work, we will extend these approaches to drug targets of complex diseases.

It has been demonstrated that diabetic pathway biomarkers can be identified from gene expression profiling data [51]. Therefore, in future work, we will focus on integrating disease-related signaling networks in the network controllability study and designing methods for identifying drug targets and biomarkers for specific genes. In addition, other multilayer nonlinear controllability objectives, such as an improved algorithm for larger multilayer networks, could be explored in future work. In addition, other meaningful node information, such as drugs specificity and particular node functions could be considered for identifying meaningful MFVS for multilayer networks.

## Conclusions

In this study, we have presented a computational framework for detecting the driver nodes in multilayer networks and applied to three real networks. The obtained results suggest that our proposed method could be a promising tool for higher drug target enrichment or discovering more meaningful steering nodes. This work can provide a good foundation for exploring and analyzing complex networks based on big data sets.

## Methods

### Mathematical model of nonlinear multilayer networks

**Fig. 5:**
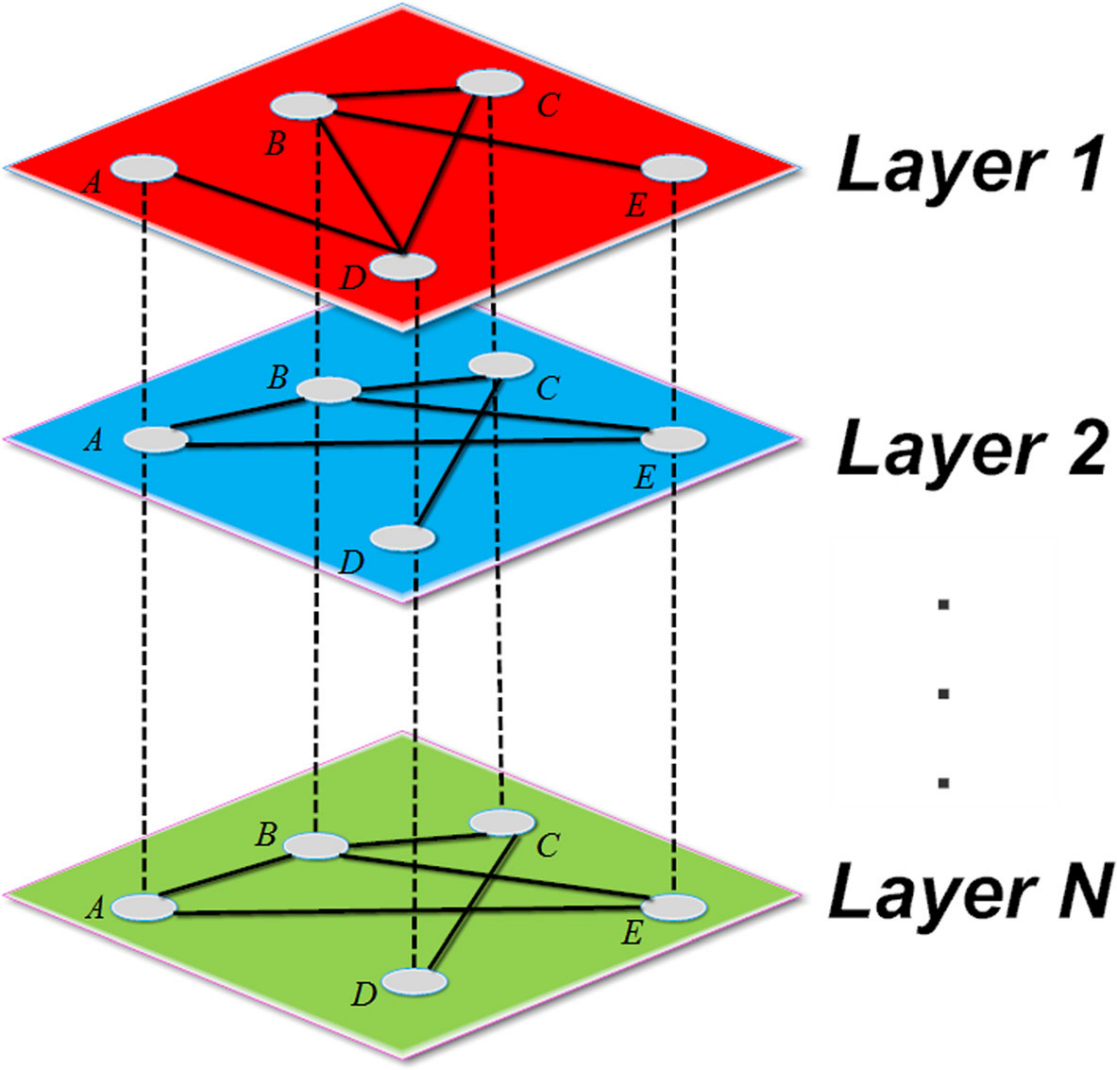
The* N*-layer network shares the same set of nodes and different interactions

Most real-world biological systems are multilayer networked structures characterized by different types of interrelationships and extremely complicated nonlinear processes. In this study, we focus on nonlinear multiplayer networked systems, which consist of a fixed set of nodes connected by different types of links (Fig. 5) and each layer is a complicated nonlinear dynamical process. To explore the controllability of multilayer networks with nonlinear dynamics, a system with *S* layers and *N* nodes can be described with the following ordinary differential equations (ODEs):

1 $$ \overset{\cdotp }{x(t)}=F(x)+ Bu(t) $$

Where *x*(*t*) = [*x*^(1)^, *x*^(2)^, ⋯, *x*^(*S*)^]^*T*^ and ${x}^{\left(\alpha \right)}={\left({x}_1^{\left(\alpha \right)},{x}_2^{\left(\alpha \right)},\cdots, {x}_N^{\left(\alpha \right)}\right)}^T$ (*α* = 1, 2, ⋯, *S*) represent the state of nodes in layer *α*. *F*(*x*) = [*F*^(1)^(*x*)  …  *F*^(*S*)^(*x*)]^*T*^ and ${F}^{\left(\alpha \right)}={\left({F}_1^{\left(\alpha \right)},{F}_2^{\left(\alpha \right)},\cdots, {F}_N^{\left(\alpha \right)}\right)}^T$ (*α* = 1, 2, ⋯, *S*) is a continuously nonlinear differentiable vector function for layer *α*. *B* =  *diag* (*B*^(1)^, *B*^(2)^, ⋯, *B*^(*S*)^) ∈ℝ^*NS* × *M*^ is the control matrix and *B*^(*β*)^(*β* = 1, 2, ⋯, *S*) are the *N* × *P*^*β*^ matrices describing the coupling between the nodes of each layer *α* and *P*^*β*^ ≤ *N* external signals.

The controllability of nonlinear multilayer networks was studied under the assumption that driver nodes are the same in all layers, which mimics the situation in which input nodes can send different signals in the different layers of the multiplex but the position of the external signals in the layers is correlated. In this study, the objective of control problems for multilayer networks with nonlinear dynamics is to identify the minimal driver nodes in the system (1) that steer this multilayered nonlinear dynamical system toward any desired dynamical attractor of each layer.

### The minimum union optimization model for controlling nonlinear multilayer networks

In this section, we present a minimum union optimization model for identifying the minimum set of driver nodes that steer the system (1) toward any desired dynamical attractor of each layer.

**Fig. 6:**
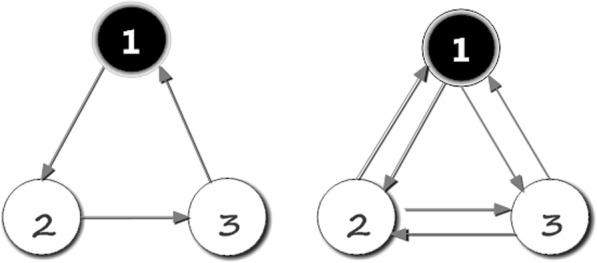
Schematic illustration of FVS in single-layer networks. The black vertices are one choice for a minimal feedback vertex set in two single-layer networks. The feedback vertices set is a subset of vertices in a directed graph, such that the removal of the set leaves the graph without directed cycles

In the nonlinear single-layer networks, Fiedleret al. [14] showed that controllability of a single network can be mapped to the feedback vertex set problem. The explanation of FVS is shown in Fig. 6. In real multilayer networks, however, nodes are usually univocally defined and share common properties across different layers, therefore, we made the assumption that each node of the multilayer network is either a driver node in each layer or it is not a driver node in any layer.

Based on the above assumption, the problem of finding the minimal set of driver nodes in the nonlinear multilayer network can be thus formulated as a minimum FVS union optimization problem, which can drive the nonlinear multilayer networked system from an arbitrary initial state to any desired dynamical attractor. Mathematically, we formulate the nonlinear control of multilayer networks as a minimum FVS union optimization problem as follows:

2 $$ \min {F}_1\cup {F}_2\cup \dots \cup {F}_n $$

**Fig. 7:**
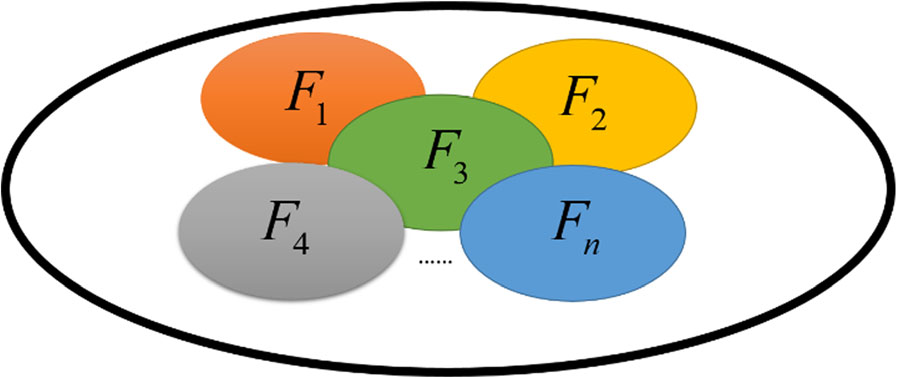
Illustration of the minimum FVS union optimization problem in a multilayer network. Suppose that *F*_1_, *F*_2_ ,…, *F*_*N*_ is the FVS in layer 1, layer 2, …, layer *N* respectively. The problem is converted to find the minimal union of *F*_1_, *F*_2_ ,…, *F*_*N*_, namely, min*F*_1_ ∪ *F*_2_ ∪  …  ∪ *F*_*N*_

Where *F*_*i*_ is the number of FVS in layer *i*, which is a set of driver nodes of the layer *i*(*i* = 1 ,2,…, *N*). The model can be illustrated as shown in Fig. 7.

### The algorithm for solving the minimum union optimization model

Currently, several algorithms have been applied to investigate the MFVS in a single-layer network. Razgon [52] proposed the GetMAS algorithm to find the MFVS of a directed graph in *O*(2^*n*^). To save time, Cai X. [53] investigated simplification of a directed graph when calculating the MFVS. We combined the two concepts and proposed the simplified GetMAS (SG) algorithm to quickly obtain the MFVS of single-layer networks. Thus, the MFVS of a multilayer network can be solved as follows:

- 1)Find the feedback vertex set of each layer with the SG algorithm.
- 2)Get an intersection of the above sets.

We named the method of getting the intersection of FVS directly as the intersection simplification GetMAS (ISG) algorithm. However, the feedback vertex set from the ISG algorithm is not a minimal set of driver nodes. The algorithm presented in this study was meant to optimize the ISG algorithm.

The improved ISG (IISG) algorithm based on a greedy principle was proposed to identify the MFVS of multilayer networks with *N*(*N* ≥ 2) layers. Before introducing the algorithm, we define three following concepts.

- *Definition 1*:(*Reference layer*): The reference layer is the layer for which we first obtain the FVS with the SG algorithm.
- *Definition 2*:(*Study layer*): The study layer is the layer for which we obtain the FVS with the IISG algorithm.
- *Definition 3*:(*Dominated nodes*): Dominated nodes consists of three kinds of nodes: nodes with more loops, nodes in the FVS of the reference layer and nodes with more functions.

Therefore, the IISG algorithm for finding the MFVS can be described in the following steps:

- Step 1: In the reference layer, the set *F*_*r*_ (the set of reference layer FVS nodes) is obtained with the SG algorithm.
- Step 2: In the study layer, calculate *L*_*i*_(*i* = 2, 3, …, *N*) (the set of the passing loops of all nodes in set *F*_*r*_).
- Step 3: In the study layer, choose the dominated nodes and remove them from the study layer to set *F*_*i*_(*i* = 2, 3, …, *N*) (which is the set of study layer FVS nodes). Calculate *L*_*i*_(*i*=2,3,...,*N*).
- Step 4: Judge whether the set *L*_*i*_(*i* = 2, 3 ,…, *N*) is empty or not. If it is an empty set, execute step 5; otherwise, repeat Steps 2 and 3.
- Step 5: In the study layer, calculate *L*_*i*_^′^(*i* = 2, 3, …, *N*) (the collection of all the passing loops of the current nodes in the study layer after removing dominated nodes from the study layer to set *F*_*i*_(*i* = 2, 3, …, *N*)).
- Step 6: In the study layer, choose the dominated node sand remove them from the study layer to set *F*_*i*_(*i* = 2, 3, …, *N*). Calculate *L*_*i*_'(*i*=2,3,...,*N*).
- Step 7: Judge whether the set *L*_*i*_^′^(*i* = 2, 3, …, *N*) is empty or not. If it is an empty set, execute step 8; otherwise, repeat Steps 5 and 6.
- Step 8: *F*_1, 2, …, i_ (the minimal driver nodes of a multilayer network where there is a multiplex with *N* = *i* layer) is the union of *F*_*r*_ and *F*_*i*_(*i* = 2, 3, …, *N*).

In particular, when the number of layers in a multilayer network exceeds two, the set *F*_1, 2, …, i_ is regarded as the set *F*_*r*_, and then we can start the next calculation.

The pseudocode of the IISG algorithm is presented as follows. .

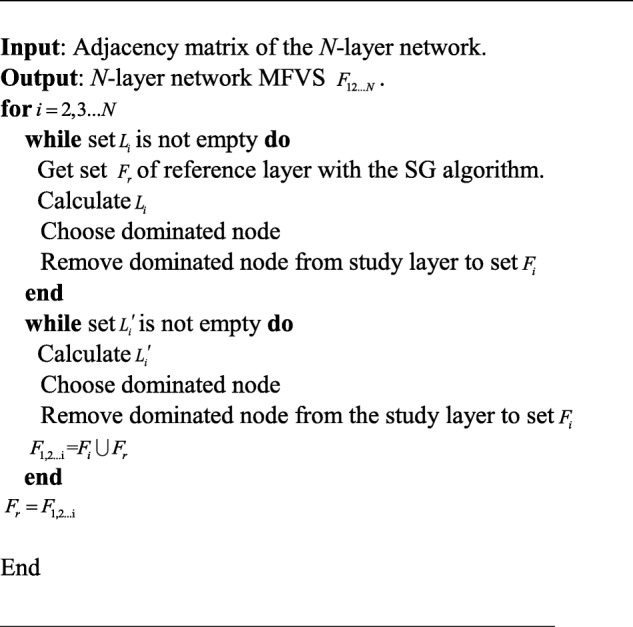



Where *F*_*r*_ is the set of reference layer FVS nodes with the SG algorithm and *L*_*i*_(*i* = 2, 3, …, *N*) is the set of the passing loops of all nodes in set *F*_*r*_ in the study layer. *F*_*i*_(*i* = 2, 3, …, *N*) is the set of study layer FVS nodes. *L*_*i*_^′^(*i* = 2, 3, …, *N*) is the collection of all the passing loops of the current nodes in the study layer after removing the dominated nodes from the study layer to set *F*_*i*_. Set *F*_12, …, n_ is the minimal driver nodes of the *N*-layer network.

**Fig. 8:**
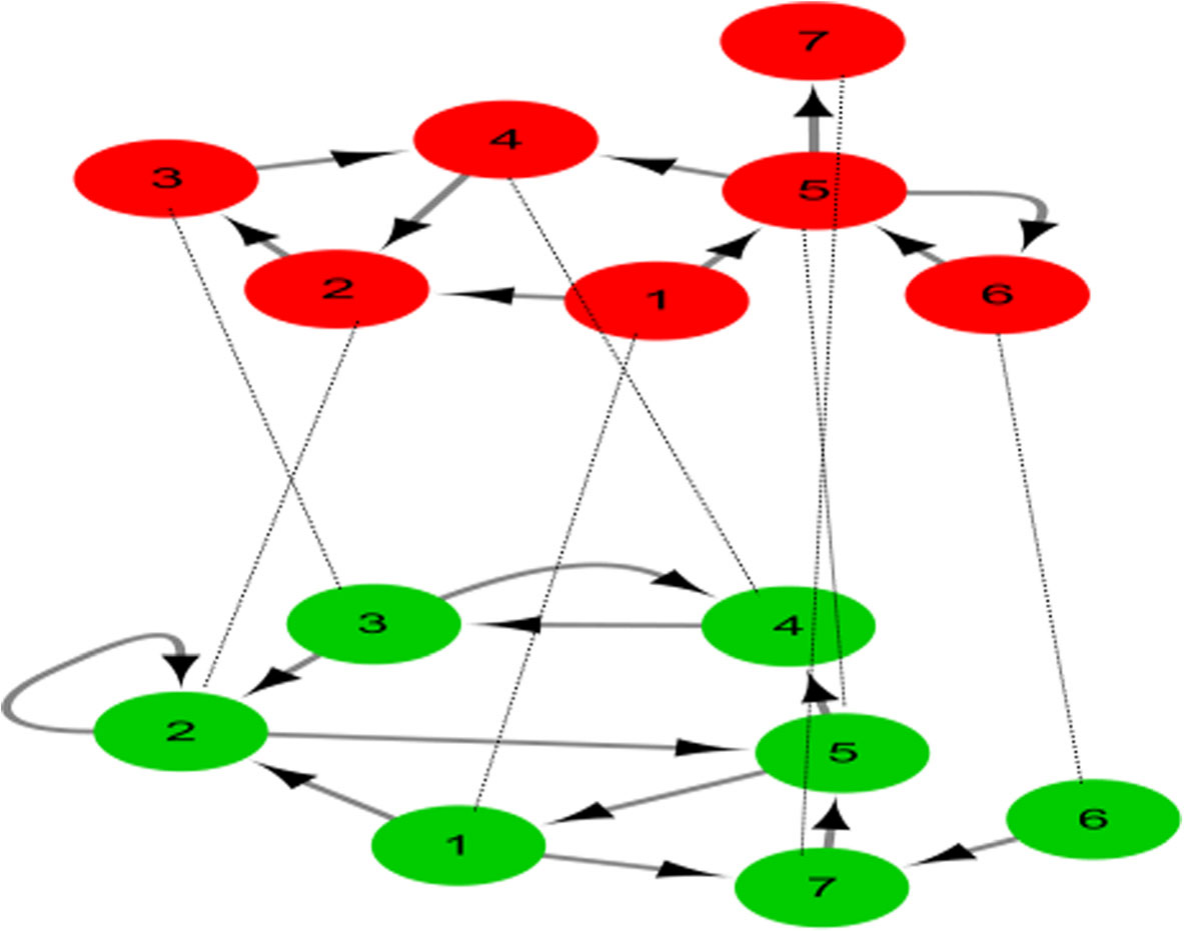
Control of a duplex network (multiplex with *N* = 2 layers). The layer with red nodes is the reference layer and the layer with green nodes is the study layer

To further illustrate the IISG algorithm, we consider a simple duplex network in Fig. 8. Because there is no biological process involved in this example, dominated nodes with more biological functions were not considered when the dominated nodes were selected. The nodes numbered 2 and 5 are one choice for MFVS in the reference layer (the layer with red nodes in Fig. 8). The 4 circles, 2, 2; 1, 2, 5; 2, 5, 4, 3 and 1, 5, 7, were found in the study layer (the layer with green nodes in Fig. 8). Nodes 2 and 5 pass through the most circles (3 circles) 2, 2; 1, 2, 5; 2, 5, 4, 3 and 1, 2, 5; 2, 5, 4, 3; 1, 5, 7, respectively, and there are also nodes in the reference layer FVS. Therefore, we consider nodes 2 and 5 as the dominated nodes and as members of FVS in the study layer. Next, they are removed from the study layer. In the study layer, there were only nodes 1, 3, 4, 6 and 7, which contained only one circle for 3 and 4. If it is a specific biological problem, we can choose a node as the FVS according to the biological function of nodes 3 and 4. In this study, we temporarily selected node 3. In conclusion, the FVS of the reference layer contains nodes 2 and 5 and the FVS of the study layer contains nodes 2, 5 and 3. Therefore, the MFVS of the duplex network contains nodes 2, 5 and 3.

## Additional file

Additional file 1: **Figure S1.** In Pathways in cancer, the above genes are clustered near the PI3K-AKT signaling pathway. **Figure S2.** In Colorectal cancer pathway, the above genes are clustered near the PI3K-AKT signaling pathway and MAPK signaling pathway. **Figure S3.** In Apoptosis pathway, the above genes are clustered near the PI3K-AKT signaling pathway and NF-κB signaling pathway. **Figure S4.** In B cell receptor signaling pathway, the above genes are clustered near the PI3K-AKT signaling pathway, MAPK signaling pathway and NF-κB signaling pathway. **Figure S5.** In T cell receptor signaling pathway, the above genes are clustered near the MAPK signaling pathway and NF-κB signaling pathway. **Figure S6.** The enrichment analysis of genes from MsigDB database. **Table S1.** Biological functions of nodes in MFVS for three multilayer networks. **Table S2.** The names of drugs that AKT, P21, BCATENIN, IFNG, JAK, JUN, NFKB, IKB, PI3K, RAF, SMAD, P53 and IAP can combine with. **Table S3.** The drugs which ENV, GAG, NEF and GAG-POL can combine with**. Table S4.** Enrichment analysis of genes in MFVS of the HHMG network. **Table S5.** The biological information of cytokines and cell types related to cancer immune system. (PDF 626 kb)
