# Supplementary material for: Control of multilayer biological networks and applied to target identification of complex diseases
Source: BMC Bioinformatics. 2019 May 28;20:271. doi: 10.1186/s12859-019-2841-2 (PMC6540418; doi:10.1186/s12859-019-2841-2)
Supplement: Supplementary file 1 — Figure S1. In Pathways in cancer, the above genes are clustered near the PI3K-AKT signaling pathway. Figure S2. In Colorectal cancer pathway, the above genes are clustered near the PI3K-AKT signaling pathway and MAPK signaling pathway. Figure S3. In Apoptosis pathway, the above genes are clustered near the PI3K-AKT signaling pathway and NF-κB signaling pathway. Figure S4. In B cell receptor signaling pathway, the above genes are clustered near the PI3K-AKT signaling pathway, MAPK signaling pathway and NF-κB signaling pathway. Figure S5. In T cell receptor signaling pathway, the above genes are clustered near the MAPK signaling pathway and NF-κB signaling pathway. Figure S6. The enrichment analysis of genes from MsigDB database. Table S1. Biological functions of nodes in MFVS for three multilayer networks. Table S2. The names of drugs that AKT, P21, BCATENIN, IFNG, JAK, JUN, NFKB, IKB, PI3K, RAF, SMAD, P53 and IAP can combine with. Table S3. The drugs which ENV, GAG, NEF and GAG-POL can combine with. Table S4. Enrichment analysis of genes in MFVS of the HHMG network. Table S5. The biological information of cytokines and cell types related to cancer immune system. (PDF 626 kb) [file 12859_2019_2841_MOESM1_ESM.pdf]

Additional file 1 for Control of multilayer biological networks and applied to target identification of complex diseases

Wei Zheng, Dingjie Wang, Xiufen Zou

Additional Figures

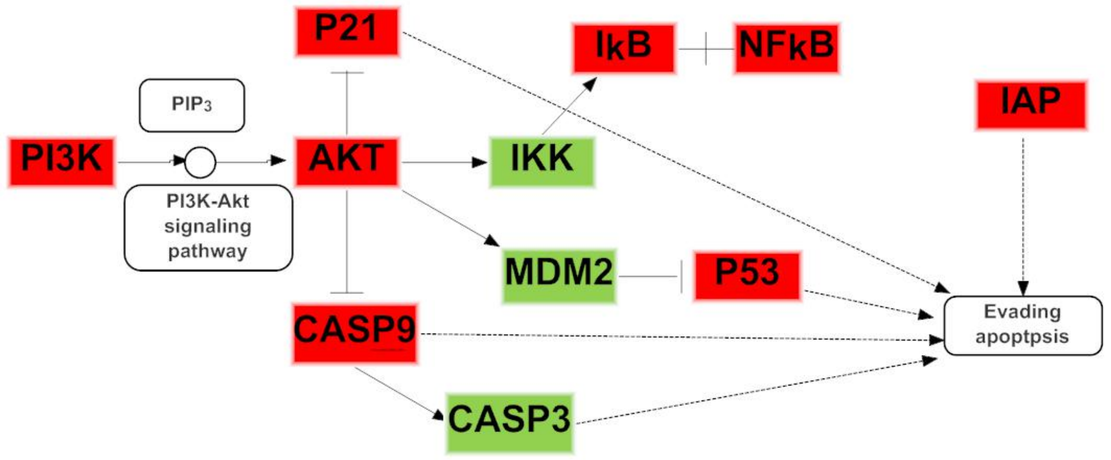

Fig.S1. In Pathways in cancer, the above genes are clustered near the PI3K-AKT signaling pathway. The red genes are the genes in MFVS of the CACC network, while the genes with other colors are not.

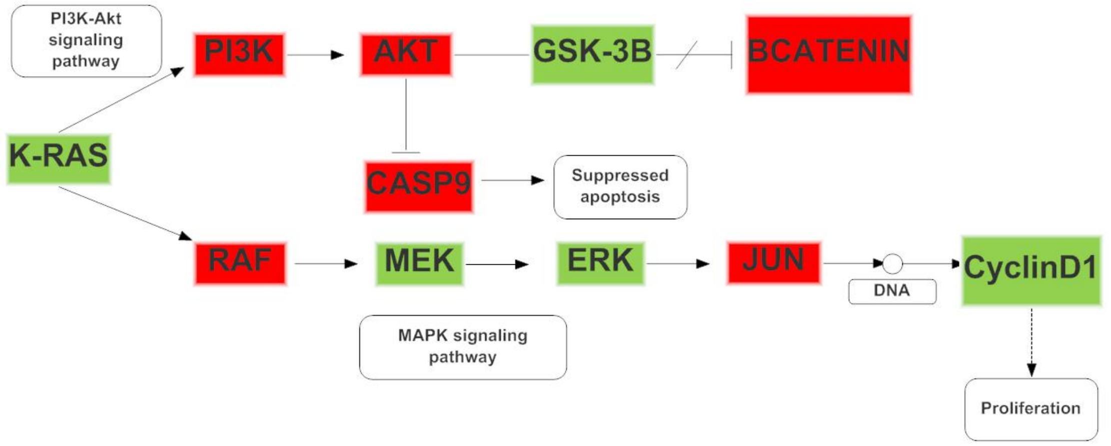

Fig. S2. In Colorectal cancer pathway, the above genes are clustered near the PI3K-AKT signaling pathway and MAPK signaling pathway. The red genes are the genes in MFVS of the CACC network, while the other color nodes are not.

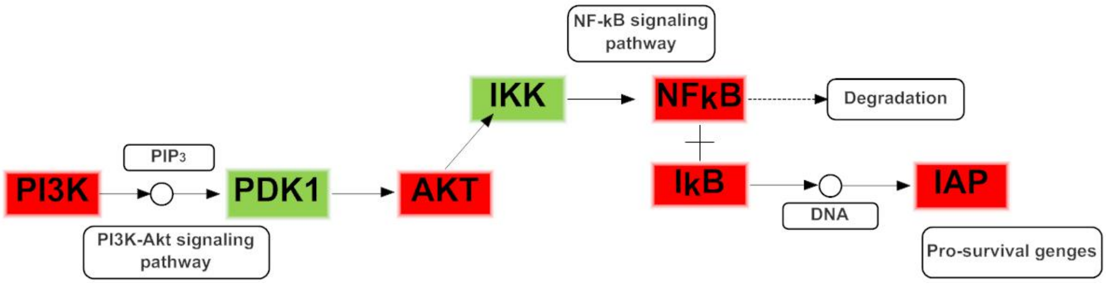

Fig. S3. In Apoptosis pathway, the above genes are clustered near the PI3K-AKT signaling pathway and NF-κB signaling pathway. The red genes are the genes in MFVS of the CACC network, while the other color nodes are not.

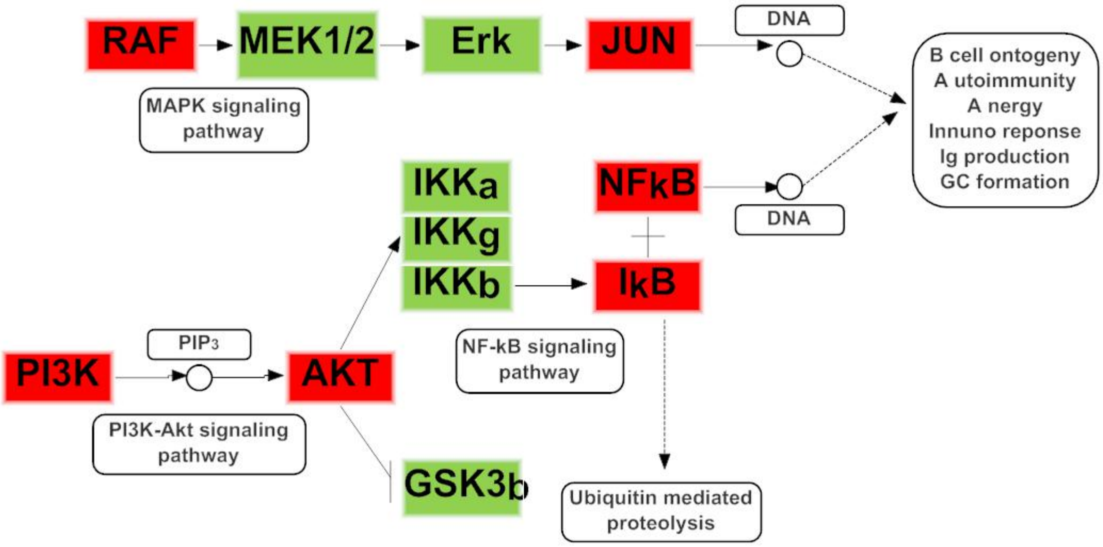

Fig. S4. In B cell receptor signaling pathway, the above genes are clustered near the PI3K-AKTsignaling pathway, MAPK signaling pathway and NF-κB signaling pathway. The red genes are the genes in MFVS of the CACC network, while the other color nodes are not.

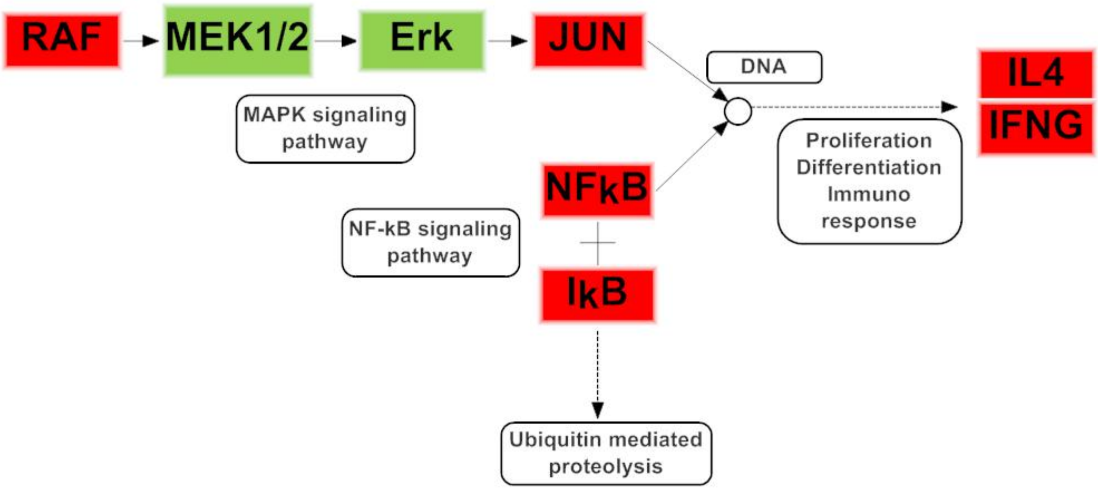

Fig. S5. In T cell receptor signaling pathway, the above genes are clustered near the MAPK signaling pathway and NF-κ B signaling pathway. The red genes are the genes in MFVS of the CACC network, while the other color nodes are not.

	KEGG_PATHWAYS_IN_CANCER	KEGG_COLORECTAL_CANCER	GO_NEGATIVE_REGULATION_OF_CELL_DEATH	KEGG_PANCREATIC_CANCER	GO_REGULATION_OF_CELL_DEATH	KEGG_CHRONIC_MYELOID_LEUKEMIA	GO_POSITIVE_REGULATION_OF_PROTEIN_METABOLIC_PROCESS	KEGG_SMALL_CELL_LUNG_CANCER	KEGG_APOPTOSIS	KEGG_PROSTATE_CANCER
AKT1	■	■	■	■	■	■	■	■	■	■
AKT2	■	■	■	■	■	■	■	■	■	■
PIK3CA	■	■	■	■	■	■	■	■	■	■
TP53	■	■	■	■	■	■	■	■	■	■
RAF1	■	■	■	■	■	■	■			■
SMAD4	■	■	■	■	■	■	■			
SMAD3	■	■	■	■	■	■	■			
CTNNB1	■	■	■		■		■			■
JUN	■	■	■		■		■			
CASP9	■	■		■	■		■	■	■	■
PIK3CB	■	■		■		■	■	■	■	■
SMAD2	■	■		■						
RELA	■		■	■	■	■	■	■	■	■
NFKB1	■		■	■	■	■		■	■	■
NFKBIA	■		■		■	■	■	■	■	■
CDKN1A	■		■		■	■	■			■
BIRC2	■		■		■		■	■	■	■
BIRC3	■		■		■		■	■	■	
XIAP	■		■		■		■	■	■	
NFKB2	■									
IL4			■		■		■			
JAK2			■		■		■			
SPHK1			■		■		■			
IFNG					■		■			
NFKBIB										

Fig. S6. The enrichment analysis of genes from MsigDB database. Based on the MSigDB database, the identified pathways are sorted by p-value from small to large and the top 10 pathways are selected as the abscissa. The ordinate represents the genes contained in the set of driver nodest of the CACC network. The blue table indicates that the genes is in the pathway

and the white table indicates the absence.

Additional Tables

Table S1 Biological functions of nodes in MFVS for three multilayer networks

CACC network		HHMG network		CICC network	
**MFVS**	**Functions**	**MFVS**	**Functions**	**MFVS**	**Functions**
**AKT**	drug target	**ENV**	drug target	**TGFß**	TGFß is considered to be a critical suppressor of T cell activities which kill the tumor cells directly. TGFß leads to cancer proliferation.
**P21**	drug target	**GAG POL**	drug target	**Cancer cells**	Cancer cell is the major target we would like to investigate.
**BCAT ENIN**	drug target	**GAG**	drug target	**NK cells**	The NK cell, by boosting the immune system and restricting the growth of tumors, is considered to be one of the major inhibitors of tumor cells. Increasing the proliferations of NK cells is an anticancer strategy.
**IFNG**	drug target	**NEF**	drug target	**CD 4 T cells**	CD4 T cell is one of the inhibiting cell types of cancer. CD4 T cells are an equally critical component of the antitumor immune response. Successful immunity to cancer therefore requires activation of tumor-specific CD4 T cells.
**JAK**	drug target	**VPU**	Vpu , an accessory protein which is encoding by HIV-1 ,is involved in several immunomodulatory functions, including counteraction of the host	**DC**	By promoting the activity of CD8, CD4 and TREG cells, dendritic cells (DC) provide major mechanisms for T-cell activation.

			restriction factor tetherin, downmodulation of CD4 T cells.	
**JUN**	drug target	**REV**	In addition to controlled processing of RNA， HIV-1 replication is also dependent on the activities provided Tat and Rev encoding by HIV-1.	
**NFKB**	drug target	**VIF**	Vif, HIV-1 accessory protein , is necessary for the production of infectious virions by CD4 lymphocytes.	
**IKB**	drug target	**VPR**	HIV-1 encodes vpr, a 96-amino-acid protein, which can prevent proliferation of infected cells by acting as a primarily cytostatic.	
**PI3K**	drug target	**TAT**	It has been revealed that the mechanism of Tat activation of RNA Polymerase II elongation of the integrated HIV-1.	
**RAF**	drug target			
**SMAD**	drug target			
**P53**	drug target			
**IAP**	drug target			.
**CASP9**	Based on STITCH database, CASP9 can interact with 4 chemicals: cisplatin, 15d-PGJ2, cordycepin, and hydrogen perox, out of which cisplatin is a platinum-based chemotherapy drug used to treat various types of cancers, including sarcomas, some carcinomas (e.g. small cell lung cancer, and ovarian cancer), lymphomas and germ cell tumors.			.
**IL4**	Based on STITCH database, IL4			

	can interact with 4 chemicals: montelukast, ALLERGENS, retinoic acid, and tacrolimus, out of which tacrolimus is an immunosuppressive drug and retinoic acid is also a medication used for the treatment of some certain cancers.	
**SPHK1**	SPHK1 plays an important role in tumorigenesis, hormonal therapy, chemotherapy resistance, and it is regarded as a new target for cancer therapeutics.	
**TREG**	TREG is regulatory T cell which is the major components for immunosuppression by promoting suppressive cytokines and inhibiting effector T cells (CD8 T cells and NK cells) directly in the cancer-immune system.	

Table S2 The names of drugs that AKT, P21, BCATENIN, IFNG, JAK, JUN , NFKB, IKB, PI3K, RAF,SMAD, P53 and IAP can combine with

**Drug Target**	**AKT(10)**	**P21(1)**	**BCATENIN (1)**	**IFNG (9)**	**JAK (6)**	**JUN (4)**	**NFKB (9)**	**IKB (3)**	**PI3K (2)**	**RAF (8)**	**SMAD (1)**	**P53(7)**	**IAP (4)**
Drugs	Arsenic trioxide	Arsenic trioxide	Urea	Glucosamine	Ruxolitinib	LGD-1550	P54	Bardoxolone methyl	Caffeine	Sorafenib	Dexfosfoserine	Acetylsalicylic acid	AEG35156
	Resveratrol			Olsalazine	Baricitinib	Vinblastine	SGN-30	Astaxanthin	XL765	LErafAON		Zinc	Dequalinium
	N-[2-(5-methyl-4H-1,2,4-triazol-3-yl)phenyl]-7H-pyrrolo[2,3-d]pyrimidin-4-amine			Foreskin keratinocyte (neonatal)	2-(1,1-DIMETHYLETHYL)9-FLUORO-3,6-DIHYDRO-7H-BENZ[H]-IMIDAZ[4,5-F]ISOQUINOLI	Irbesartan	Thalidomide	Acetylsalicylic acid		Regorafenib		1-(9-ethyl-9H-carbazol-3-yl)-N-methylmethanamine	N-METHYLALANYL-3-METHYLVALYL-4-PHENOXY-N-(1,2,3,4-TETRAHYDRONAPHTHALEN-

					N-7-ONE								1-YL)PROLINAMIDE
	5-(5-chloro-7H-pyrrolo[2,3-d]pyrimidin-4-yl)-4,5,6,7-tetrahydro-1H-imidazo[4,5-c]pyridine			Foreskin fibroblast (neonatal)	Tofacitinib	Arsenic trioxide	HE3286			Fostamatinib		Triethyl Phosphate	1-[3,3-Dimethyl-2-(2-Methylamino-Propionylamino)-Butyryl]-Pyrrolidine-2-Carboxylic Acid(1,2,3,4-Tetrahydro-Naphthalen-1-Yl)-Amide
	ATP			Apremilast	Fostamatinib		NOX-700			XL281		AZD 3355	
	Genistein			Fontolizumab	Myricetin		Custirsen			iCo-007		Zinc acetate	
	Perifosine			VIR201			Pranlukast			Cholecystokinin		Zinc chloride	
	Archexin			Emapalumab			Andrographolide			Dabrafenib			
	Enzastaurin			Interferon gamma-1b			Triflusal						
	Inositol 1,3,4,5-Tetrakisphosphate												

Table S3 The drugs which ENV, GAG, NEF and GAG-POL can combine with

Drug Target	ENV(8)	GAG (3)	NEF (1)	GAG-POL(130)			
Drugs	Astodrimer	PA-1050040	Myristic acid	Cacodylic acid	GSK-364735	Talviraline	Bevirimat
	Beta-3-Serine	Myristic acid		DMP450	Quinaldic Acid	Skf 107457	KP-1461
	2,4-Diaminobutyric Acid	Isopropyl alcohol		XV638	C31G	L-756,423	Inhibitor Bea388
	3-Amino-Alanine			SD146	Etravirine	Emivirine	Calanolide A
	Alpha-L-Fucose			AHA047	VGV-1	Myristic acid	RDEA806

	Biphenylalanine	A-98881	Dapivirine	TI-3-093	Capravirine
	N-acetyl-alpha-D-glucosamine	Inhibitor BEA369	Inhibitor Bea322	1-Benzyl-(R)-Propylamine	Analogue of Indinavir Drug
	Beta-3-Cysteine	Cystein-S-Yl Cacodylate	Inhibitor Bea403	Tert-Butyloxycarbonyl Group	NAPHTHYLOXYACETIC ACID
		3-(Mercaptomethylene)Pyridine	Alpha-Aminobutyric Acid	Inhibitor Bea425	S-(Dimethylarsenic)Cysteine
		Inhibitor Bea409	Inhibitor Bea428	UC-781	Inhibitor Msa367
		5-bromo-3-(pyrrolidin-1-ylsulfonyl)-1H-indole-2-carboxamide	(2,6-DIMETHYL-PHENOXY)-ACETIC ACID	(2S)-2-amino-3-phenylpropane-1,1-diol	3(S)-AMINO-4-PHENYL-BUTAN-2(S)-OL
		6,11-DIHYDRO-11-ETHYL-6-METHYL-9-NITRO-5H-PYRIDO[2,3-B][1,5]BENZODIAZEPIN-5-ONE	S-{2-[(2-chloro-4-sulfamoylphenyl)amino]-2-oxoethyl} 6-methyl-3,4-dihydroquinoline-1(2H)-carbothioate	6-CHLORO-2-(1-FURO[2,3-C]PYRIDIN-5-YL-ETHYLSULFANYL)-PYRIMIDIN-4-YLAMINE	1-METHYL ETHYL 2-CHLORO-5-[[[(1-METHYLETHOXY)THIOOXO]METHYL]AMINO]-BENZOATE
		2,4-DIAMINO-1,5-DIPHENYL-3-HYDROXYPENTANE	[4-R-(4-ALPHA,6-BETA,7-BETA)]-HEXAHYDRO-5,6-DI(HYDROXY)-1,3-DI(ALLYL)-4,7-BISPHENYLMETHYL)-2H-1,3-DIAZEPINONE	5-AMINO-6-CYCLOHEXYL-4-HYDROXY-2-ISOPROPYL-HEXANOIC ACID	3-[2-bromo-4-(1H-pyrazolo[3,4-c]pyridazin-3-ylmethyl)phenoxy]-5-methylbenzonitrile
		N-1H-imidazol-2-yl-N'-[4-(1H-imidazol-2-ylamino)phenyl]benzene-1,4-diamine	2-aminoethyl naphthalen-1-yl acetate	(2S)-2-amino-3-phenylpropane-1,1-diol	O-[2-(1,3-dioxo-1,3-dihydro-2H-isoindol-2-yl)ethyl] (4-bromophenyl)thiocarbamate
		(2E)-3-{3-[(5-ETHYL-3-IODO-6-METHYL-2-OXO-1,2-DIHYDROPYRIDIN-4-YL)OXY]PHENYL}ACRYLONITRILE	1-(5-Chloroindol-3-Yl)-3-Hydroxy-3-(2h-Tetrazol-5-Yl)-Propenone	4-[(CYCLOPROPYLETHYNYL)OXY]-6-FLUORO-3-ISOPROPYLQUINOLIN-2(1H)-ONE	O-[2-(1,3-dioxo-1,3-dihydro-2H-isoindol-2-yl)ethyl] (4-chlorophenyl)thiocarbamate

		(2-ACETYL-5-METHYLANILINO)(2,6-DIBROMOPHENYL)ACETAMIDE	INHIBITOR Q8467 OF DUPONT MERCK	1-(4-CYANO-PHENYL)-3-[2-(2,6-DICHLORO-PHENYL)-1-IMINO-ETHYL]-THIOUREA	NAPHTHYLOXYACETIC ACID
		ALPHA-(2,6-DICHLOROPHENYL)-ALPHA-(2-ACETYL-5-METHYLANILINO)ACETAMIDE	6-(cyclohexylsulfanyl)-1-(ethoxymethyl)-5-(1-methylethyl)pyrimidine-2,4(1H,3H)-dione	6-CHLORO-4-(CYCLOHEXYLOXY)-3-ISOPROPYLQUINOLIN-2(1H)-ONE	1-[2-(4-ETHOXY-3-FLUOROPYRIDIN-2-YL)ETHYL]-3-(5-METHYLPYRIDIN-2-YL)THIOUREA
		1-(5-BROMO-PYRIDIN-2-YL)-3-[2-(6-FLUORO-2-HYDROXY-3-PROPIONYL-PHENYL)-CYCLOPROPYL]-UREA	5-ETHYL-3-[(2-METHOXYETHYL)METHYLAMINO]-6-METHYL-4-(3-METHYLBENZYL)PYRIDIN-2(1H)-ONE	(E)-3,4-DIHYDROXY-N'-[(2-METHOXYNAPHTHALEN-1-YL)METHYLENE]BENZOHYDRAZIDE	6-(4-chloro-2-fluoro-3-phenoxybenzyl)pyridazin-3(2H)-one
		(R)-(+)5(9BH)-OXO-9B-PHENYL-2,3-DIHYDROTHIAZOLO[2,3-A]ISOINDOL-3-CARBOXYLIC ACID METHYL ESTER	{(1s)-1-Benzyl-4-[3-Carbamoyl-1-(1-Carbamoyl-2-Phenyl-Ethylcarbamoyl)-(S)-Propylcarbamoyl]-2-Oxo-5-Phenyl-Pentyl}-Carbamic Acid Tert-Butyl Ester	3-BENZYLOXYCARBONYLAMINO-2-HYDROXY-4-PHENYL-BUTYRIC ACID	4-(3,5-DIMETHYLPHENOXY)-5-(FURAN-2-YLMETHYLSULFANYLMETHYL)-3-IODO-6-METHYLPYRIDIN-2(1H)-ONE
		N-(4-{[amino(imino)methyl]amino}butyl)-2,4'-bi-1,3-thiazole-4-carboxamide	2-[4-(Hydroxy-Methoxy-Methyl)-Benzyl]-7-(4-Hydroxymethyl-Benzyl)-1,1-Dioxo-3,6-Bis-Phenoxymethyl-1lambda6-[1,2,7]Thiadiazepane-4,5-Diol	3-chloro-5-[2-chloro-5-(1H-pyrazolo[3,4-b]pyridin-3-ylmethoxy)phenoxy]benzonitrile	3-{5-[(6-amino-1H-pyrazolo[3,4-b]pyridin-3-yl)methoxy]-2-chlorophenoxy}-5-chlorobenzonitrile
		(9S,12S)-9-(1-methylethyl)-7,10-dioxo-2-oxa-8,11-diazabicyclo[12.2.2]o	3-(CARBOXYAMIDE(2-CARBOXYAMIDE-2-TERTBUTYLETHYL))PENTA	4-CHLORO-8-METHYL-7-(3-METHYL-BUT-2-ENYL)-6,7,8,9-TETRAHYD	4-(4-CHLORO-PHENYL)-1-{3-[2-(4-FLUORO-PHENYL)-[1,3]DITHIOLAN-2-YL]-PR

		ctadeca-1(16), 14,17-triene-12-carboxylic acid	N	RO-2H-2,7,9A-TRIAZA-BENZO[CD]AZULENE-1-THIONE	OPYL}-PIPERIDIN-4-OL
		(2R,4S)-2-[(R)-BENZYLCARBAMOYL-PHENYLACETYL-METHYL]-5,5-DIMETHYL-THIAZOLIDINE-4-CARBOXYLIC ACID	6-BENZYL-1-BENZYLOXYMETHYL-5-ISOPROPYL URACIL	3-[1-(4-BROMO-PHENYL)-2-METHYL-PROPYL]-4-HYDROXY-CHROMEN-2-ONE	(3s)-Tetrahydrofuran-3-Yl (1r,2s)-3-[4-((1r)-2-{[(S)-Amino(Hydroxy)Methyl]Oxy}-2,3-Dihydro-1h-Inden-1-Yl)-2-Benzyl-3-Oxopyrrolidin-2-Yl]-1-Benzyl-2-Hydroxypropylcarbamate
		6-(3',5'-DIMETHYLBENZYL)-1-ETHOXYMETHYL-5-ISOPROPYLURACIL	({3-[1-(4-HYDROXY-2-OXO-2H-CHROMEN-3-YL)-PROPYL]-PHENYLCARBAMOYL}-METHYL)-CARBAMIC ACID TERT-BUTYL ESTER	5,6,7,8,9,10-HEXAHYDRO-4-HYDROXY-3-(1-PHENYLPROPYL)CYCLOOCTA[B]PYRAN-2-ONE	(4r,5s,6s,7r)-1,3-Dibenzyl-4,7-Bis(Phenoxymethyl)-5,6-Dihydroxy-1,3 Diazepan-2-One
		6-CHLORO-4-(CYCLOHEXYLOXY)-3-PROPYLQUINOLIN-2(1H)-ONE	N-(3-Cyclopropyl(5,6,7,8,9,10-Hexahydro-2-Oxo-2h-Cycloocta[B]Pyran-3-Yl)Methyl)Phenylbenzensulfonamide	(3,4-Dihydroxy-Phenyl)-Triphenyl-Arsonium	Je-2147, Ag1776, Kni-764
		(2r,3r,4r,5r)-3,4-Dihydroxy-N,N'-Bis[(1s,2r)-2-Hydroxy-2,3-Dihydro-1h-Inden-1-Yl]-2,5-Bis(2-Phenylethyl)Hexanediamide	N-[2(S)-Cyclopentyl-1(R)-Hydroxy-3(R)Methyl]-5-[(2(S)-Tertiary-Butylamino-Carbonyl)-4-(N1-(2)-(N-Methylpiperazinyl)-3-Chloro-Pyrazinyl-5-Carbonyl)-Piperazino]-4(S)-Hydroxy-2(R)-Phenylmethyl-	3-{[(5R,6R)-5-Benzyl-6-hydroxy-2,4-bis(4-hydroxybenzyl)-3-oxo-1,2,4-triazepan-1-yl]sulfonyl}benzonitril	N-[2-Hydroxy-2-(8-Isopropyl-6,9-Dioxo-2-Oxa-7,10-Diaza-Bicyclo[11.2.2]Heptadeca-1(16),13(17),14-Trien-11-Yl)-Ethyl]-N-(3-Methyl-Butyl)-Benzenesulfonamide,Inhibitor 3

		3-chloro-5-[2-chloro-5-(1H-indazol-3-ylmethoxy)phenoxy]benzonitrile	Pentanamide 2-(11-{2-[Benzenesulfonyl-(3-Methyl-Butyl)-Amino]-1-Hydroxy-Ethyl}-6,9-Dioxo-2-Oxa-7,10-Diaza-Bicyclo[11.2.2]Heptadeca-1(16),13(17),14-Trien-8-Yl)-Acetamide, Inhibitor 2	(6-[4-(AMINOMETHYL)-2,6-DIMETHYLPHENOXY]-2-{[4-(AMINOMETHYL)PHENYL]AMINO}-5-BROMOPYRIMIDIN-4-YL)METHANOL	ALPHA-(2,6-DICHLOROPHENYL)-ALPHA-(2-ACETYL-5-METHYLANILINO)ACETAMIDE
		O-[2-(1,3-dioxo-1,3-dihydro-2H-isoindol-2-yl)ethyl] (4-iodophenyl)thiocarbamate	4-[4-AMINO-6-(2,6-DICHLORO-PHENOXY)-[1,3,5]TRIAZIN-2-YLAMINO]-BENZONITRILE	4-[4-AMINO-6-(5-CHLORO-1H-INDOL-4-YLMETHYL)-[1,3,5]TRIAZIN-2-YLAMINO]-BENZONITRILE	(R)-(+)9B-(3-METHYL)PHENYL-2,3-DIHYDROTHIAZOLO[2,3-A]ISOINDOL-5(9BH)-ONE
		3-[4-(2-METHYL-IMIDAZO[4,5-C]PYRIDIN-1-YL)BENZYL]-3H-BENZOTHIAZOL-2-ONE	N-({(3R,4R)-4-[(benzyloxy)methyl]pyrrolidin-3-yl}methyl)-N-(2-methylpropyl)benzenesulfonamide	N-[[3-FLUORO-4-ETHOXY-PYRID-2-YL]ETHYL]-N'-[5-NITRILOMETHYL-PYRIDYL]-THIOUREA	N-[[3-FLUORO-4-ETHOXY-PYRID-2-YL]ETHYL]-N'-[5-CHLORO-PYRIDYL]-THIOUREA
		1-[2-(4-ETHOXY-3-FLUOROPYRIDIN-2-YL)ETHYL]-3-(5-METHYLPYRIDIN-2-YL)THIOUREA	1-METHYL ETHYL 1-CHLORO-5-[[(5,6DIHYDRO-2-METHYL-1,4-OXATHIIN-3-YL)CARBONYL]AMINO]BENZOATE	2-[4-chloro-2-(phenylcarbonyl)phenoxy]-N-phenylacetamide	6-CHLORO-4-(CYCLOHEXYLSULFANYL)-3-PROPYLQUINOLIN-2(1H)-ONE
		PIPERIDINE-2-CARBOXYLIC ACID TERT-BUTYLAMIDE	N-{3-[(E)-(tert-butoxyimino)methyl]-4-chlorophenyl}-2-methylfuran-3-carbimidothioic acid	6-CHLORO-4-(CYCLOHEXYLSULFINYL)-3-PROPYLQUINOLIN-2(1H)-ONE	ISOPROPYL (2S)-2-ETHYL-7-FLUORO-3-OXO-3,4-DIHYDROQUINOXALINE-1(2H)-CARBOXYLATE
		3(S)-AMINO-4-PHENYL-BUTAN-2(S)-OL	4-HYDROXY-7-METHOXY-3-(1-PHENYL-PR	1-(2-HYDROXYETHYLOXYMETHYL)-6-P	6,7,8,9-TETRAHYDRO-4-HYDROXY-3-(1-PHEN

			OPYL)-CHROMEN-2-ONE	HENYL THIOTHYMINE	YLPROPYL)CYCLOHEPTA[B]PYRAN-2-ONE
		3-[6-bromo-2-fluoro-3-(1H-pyrazolo[3,4-c]pyridazin-3-ylmethyl)phenoxy]-5-chlorobenzonitrile	2-AMINO-6-(3,5-DIMETHYLPHENYL)SULFONYLBENZONITRILE	1-(3-chloro-4-methylphenyl)-3-{2-[({5-[(dimethylamino)methyl]-2-furyl}methyl)thio]ethyl}urea	1-[2-(3-ACETYL-2-HYDROXY-6-METHOXY-PHENYL)-CYCLOPROPYL]-3-(5-CYANO-PYRIDIN-2-YL)-THIOUREA
		5-CHLORO-8-METHYL-7-(3-METHYL-BUT-2-ENYL)-6,7,8,9-TETRAHYDRO-2H-2,7,9A-TRIAZA-BENZO[CD]AZULENE-1-THIONE	4-CHLORO-8-METHYL-7-(3-METHYL-BUT-2-ENYL)-6,7,8,9-TETRAHYDRO-2H-2,7,9A-TRIAZA-BENZO[CD]AZULENE-1-THIONE		

Table S4 Enrichment analysis of genes in MFVS of the HHMG network.

Enrichment Score: 0.3884496061567027			
Term	Count	P Value	Genes
Host-virus interaction	9	0.2	NEF, GAG-POL, REV, GAG, VPR, ENV, VPU, TAT, VIF
Virion	8	0.377777778	NEF, GAG-POL, GAG, VPR, ENV, VPU, TAT, VIF
AIDS	8	0.377777778	NEF, GAG-POL, REV, GAG, VPR, ENV, TAT, VIF
Host cytoplasm	8	0.377777778	NEF, GAG-POL, REV, GAG, VPR, ENV, TAT, VIF
RNA-binding	8	0.377777778	GAG-POL, REV, GAG, VPR, ENV, VPU, TAT, VIF
Host cell membrane	8	0.377777778	NEF, GAG-POL, REV, GAG, VPR, ENV, VPU, VIF
GO:0020002~host cell plasma membrane	8	0.377777778	NEF, GAG-POL, REV, GAG, VPR, ENV, VPU, VIF
Host membrane	8	0.377777778	NEF, GAG-POL, REV, GAG, VPR, ENV, VPU, VIF
GO:0030430~host cell cytoplasm	8	0.377777778	NEF, GAG-POL, REV, GAG, VPR, ENV, TAT, VIF
Host nucleus	8	0.377777778	GAG-POL, REV, GAG, VPR, ENV, VPU, TAT, VIF
GO:0016020~membrane	7	0.533333333	NEF, GAG-POL, REV, GAG, VPR, ENV, VIF
GO:0042025~host cell nucleus	7	0.533333333	GAG-POL, REV, GAG, VPR, VPU, TAT, VIF
Membrane	9	1	NEF, GAG-POL, REV, GAG, VPR, ENV, VPU, TAT, VIF

Table S5 The biological information of cytokines and cell types related to cancer immune system

**Cell Type**	**Cell Name1**	**Cell Name2**	**Function**	**cancer-inhibiting\promoting cell types**
cancer cell	Cancer cell	Cancer Cell	Cancer cell	cancer-promoting cell types

cell cytokine	transforming growth factor	TGFß	TGFß, which is considered to be a critical suppressor of T cells activities.	
	natural killer cells	NK cell	NK cells are the main effective killer cells for the innate immune response.	
	effector T cells	CD4	CD4 T cells are anticancer T cells, which kill the tumor cells directly.	cancer-inhibiting cell types
		CD8	CD8 T cells are the main effective killer cells for the adaptive immune response.	
	regulatory T cells	TREG	The regulatory T cells are the major components for immunosuppression by promoting suppressive cytokines and inhibiting effector T cells (CD8 and NK) directly.	cancer-promoting cell types
	dendritic cells	DC	Dendritic cells (mDCs) are antigen-presenting cells of the immune system. They trigger the adaptive responses when detecting antigens from cancer cells. Immature dendritic cells.	cancer-inhibiting cell types
	tumor-associated macrophages cells	M1TAM	M1 phenotypic macrophages are suggested to promote tumor killing by activating CD8 T cells.	cancer-inhibiting cell types
immune cell		M2TAM	M2 type macrophages are suggested to promote tumor progression and metastasis.	cancer-promoting cell types
	myeloid derived suppressor cells	MDSC	The MDSCs are suggested to promote cancer progression.	cancer-promoting cell types
	tumour-associated neutrophil cells	TAN	TAN plays an important role in tumour growth and progression	N1 type inhibits tumor growth, while N2 type promotes tumor growth and metastasis.
	T helper cells	Th1	Th1 cells activate antigen-presenting cells (APCs) and induce the production of the type of antibodies that can enhance the chances of cancer cells turning into APCs.Th1 inhibits cancer cells by activating the APCs,	cancer-inhibiting cell types
		Th2	The Th2 response triggered by cancer pro motes the growth of cancer cells/Th2 activates cancer cells by promoting their proliferation.	cancer-promoting cell types
		Th17	Th17 cells play a potent proinflammatory role in cancer microenvironments .	
